# Child-Robot Collaborative Problem-Solving and the Importance of Child's Voluntary Interaction: A Developmental Perspective

**DOI:** 10.3389/frobt.2020.00015

**Published:** 2020-02-18

**Authors:** Vicky Charisi, Emilia Gomez, Gonzalo Mier, Luis Merino, Randy Gomez

**Affiliations:** ^1^Centre for Advanced Studies, Joint Research Centre, European Commission, Seville, Spain; ^2^Music Technology Group, Universitat Pompeu Fabra, Barcelona, Spain; ^3^Service Robotics Lab, School of Engineering, Universidad Pablo de Olavide, Seville, Spain; ^4^Honda Research Institute Japan Co., Ltd., Saitama, Japan

**Keywords:** child-robot interaction, problem solving, self-initiated interaction, robotics, education

## Abstract

The emergence and development of cognitive strategies for the transition from exploratory actions towards intentional problem-solving in children is a key question for the understanding of the development of human cognition. Researchers in developmental psychology have studied cognitive strategies and have highlighted the catalytic role of the social environment. However, it is not yet adequately understood how this capacity emerges and develops in biological systems when they perform a problem-solving task in collaboration with a robotic social agent. This paper presents an empirical study in a human-robot interaction (HRI) setting which investigates children's problem-solving from a developmental perspective. In order to theoretically conceptualize children's developmental process of problem-solving in HRI context, we use principles based on the intuitive theory and we take into consideration existing research on executive functions with a focus on inhibitory control. We considered the paradigm of the Tower of Hanoi and we conducted an HRI behavioral experiment to evaluate task performance. We designed two types of robot interventions, “voluntary” and “turn-taking”—manipulating exclusively the timing of the intervention. Our results indicate that the children who participated in the voluntary interaction setting showed a better performance in the problem solving activity during the evaluation session despite their large variability in the frequency of self-initiated interactions with the robot. Additionally, we present a detailed description of the problem-solving trajectory for a representative single case-study, which reveals specific developmental patterns in the context of the specific task. Implications and future work are discussed regarding the development of intelligent robotic systems that allow child-initiated interaction as well as targeted and not constant robot interventions.

## 1. Introduction

The emergence and development of problem-solving cognitive strategies are fundamental mechanisms for human evolution. In the case of childhood, these mechanisms allow children to generate and develop novel mental representations and schemata through playful exploratory activities which gradually transform into deliberate problem solving strategies. These cognitive mechanisms are dominant in a child's development as a combination of a series of interrelated, domain general cognitive skills associated with the prefrontal cortex, such as inhibitory control, shifting, working memory, and others which appear under the umbrella term of Executive Functions (EFs). During the last few decades, EFs have gained increasing attention in developmental and educational research (Keen, 2011; Warneken et al., 2014) and often they are associated with playful and exploratory activities (Best and Miller, 2010).

One of the core elements for the development of exploratory actions is a child's curiosity and intrinsic motivation (Oudeyer and Smith, 2016; Twomey and Westermann, 2018). This allows for the child to exhibit sustained task attention and to proceed from exploratory actions to intentional ones developing the necessary planning skills. Planning, as a prototypical EF, is a high-level cognitive process, which includes goal-directed action sequencing and inhibition of competing impulses (Blakey et al., 2016). Although the growth of EFs follows a common trend, it has been indicated that their components do not develop as a unit; rather, each individual EF follows its own trajectory which might differ among individuals (Diamond, 2006; Best and Miller, 2010; Friedman and Miyake, 2017). Thus, an increasing body of research on child development and learning focuses not only on learning outcomes but on the individual differences of learning process and the transition from one developmental stage to another (Siegler and Crowley, 1991; Best and Miller, 2010; Brock and Taber, 2017).

Among the prevalent methods used for the depiction of child's developmental process is the microgenetic analysis (Piaget and Cook, 1952; Siegler and Crowley, 1991; Lavelli et al., 2005; Montes et al., 2017). Microgenetic analysis focuses on the collection of micro-behavioral data in a dense way in order to capture the emergence and the dynamicity of cognitive development. Individuals are observed over a period of developmental change and the observations are conducted before, during, and after an intervention to capture the process of change. Observed behaviors are intensively analyzed, both qualitatively and quantitatively with the aim to identify the processes that give rise to the developmental change. The microgenetic approach has been used in various contexts such as inhibitory control (Flynn et al., 2004), memory (Schlagmüller and Schneider, 2002), mathematics (Van der Ven et al., 2012), and music-making (Charisi et al., 2018).

While individual trajectories are important for the understanding of child's cognitive development, existing theories highlight the role of social interaction in child's learning (Bandura, 1971; Vygotsky, 1978; Tomasello, 1995). For young children, the development of effective strategies for problem-solving is often associated to scaffolding from the social environment (Tomasello, 1995; Cragg and Chevalier, 2012); collaboration is particularly beneficial for low-ability children when there is an ability asymmetry (Sills et al., 2016).

Based on the above-mentioned theoretical accounts and paradigms, the field of child-robot interaction has examined the ways in which robotic agents might be suitable social learning companions for children in various age-groups and in different contexts such as in second language learning (Kennedy et al., 2016; Kory-Westlund and Breazeal, 2019), in inquiry learning (Wijnen et al., 2019), handwriting learning (Lemaignan et al., 2016), story telling (Leite et al., 2017), problem-solving (Ramachandran et al., 2018), and creativity (Alves-Oliveira et al., 2017). As a recent review on social robots in education (Belpaeme et al., 2018) indicates, social robots have consistently been proved that might be helpful in immediate learning gains in the specific contexts.

However, there are also some suggestions that robots that support child learning should limit their social behavior at targeted times based on the cognitive load and the engagement of the child (Kennedy et al., 2015; Belpaeme et al., 2018). However, the majority of the existing research in the field of child-robot interaction refers to studies with imposed canonical robot interventions which do not allow children to develop their exploratory skills and exhibit self-initiated voluntary interaction. In addition to this, most of the existing work focuses only on the learning outcomes (Charisi et al., 2016) without examining the development of learning process as it occurs and the emergence of possible patterns.

Taken together, the current research in developmental psychology and educational sciences indicate the importance of child's exploratory actions as a core strategy for the development of problem-solving skills. However, existing studies that investigate the impact of social robots in child's learning have mainly focused on imposed robot interventions. As a result, one open and important question is whether a voluntary interaction associates with child's problem-solving process and performance and what are the possible emerging patterns and trajectories of problem-solving process in the case of a canonical, such as turn-taking, and on-demand robot intervention. To address this question we conducted a two conditions repeated sessions study in which children solve a problem together with a robot. The rest of the paper presents the methodology, the data analysis and results of the study, which are discussed against the existing literature.

## 2. Methodology

### 2.1. Research Question

There are many factors that influence children's problem-solving process. In the context of voluntary child-robot interaction, this study explores how the robot's intervention style of voluntary (on-demand) interaction affect child's problem-solving process and task performance in contrast to a canonincal intervention in the form of turn-taking setting.

### 2.2. Hypotheses

To address the above mentioned research question, based on the existing theoretical and empirical work we develop a set of hypotheses:

- H1: In a child-robot interaction problem-solving activity, children that voluntarily interact with a robot are more likely to show better performance and improvements in their performance than children who interact in a turn-taking setting. We expect that because children that voluntarily interact with the robot might have more opportunities for exploration in problem-solving process.
- H2: Children who voluntary interact and who faced more difficulties in problem-solving (e.g., younger children) would ask more frequently for help by a present robot. We expect this because children in challenging situations look to learn from others (Vygotsky, 1978; Gelman, 2009).
- H3: In developmental problem-solving tasks, it is likely that patterns of solution strategies emerge over time. We expect that because of prior work on child's construct emergence (Gerstenberg and Tenenbaum, 2017).

### 2.3. Research Design

A behavioral exploratory study is designed to illustrate the relationship between child-robot voluntary interaction and child's problem-solving process and performance, focusing on the importance of exploration. In addition to being one of the first studies that implement child's voluntary interaction and exploration while interacting with robots, this study adopts the developmental design of a microgenetic approach, which allows for patterns of child's problem solving process to emerge and involves the understanding of the “how” of the learning process rather than only its outputs. This involves studying change while it is occurring (Siegler and Crowley, 1991).

The micro-genetic approach is characterized by (i) observations that span a period of rapidly changing competence; (ii) the density of observation within this period is high, relative to the rate of change of the knowledge or skills of interest; and (iii) the observations of changing performance are analyzed intensively, with the goal of inferring the representations and processes that gave rise to them. For this reason, the sample size in microgenetic approaches is typically small and possible comparisons among conditions are approached mainly in a descriptive and qualitative manner. The activities that are designed for microgenetic analysis are characterized by spontaneous and exploratory actions which gradually transform into organized and deliberate behavioral manifestations and contribute to the transition from sensori-motor to symbolic representations (Siegler and Crowley, 1991).

The current educational literature is in consensus about the role of exploration as one of the fundamental processes of child's problem solving in contrast to guided instruction (Dewey, 1902; Whitebread et al., 2012). For this reason, the study follows a two-condition design to contrast guided intervention with child's self-initiated interaction with the robot.

We manipulate robot's intervention as follows: (i) in condition 1 (Cond1), the child is instructed to solve the task in collaboration with the robot in a “turn-taking” scenario, which results in a canonical cognitive intervention by the robot and (ii) in Condition 2 (Cond2) the child is instructed to solve the task independently, having the option to ask the help of the robot whenever (if) this is needed, which results in an “on demand” intervention by the robot. Thus, the children have a self-initiative role and they are free to select if and when the robot would contribute to the solution of the task.

**Figure 1:**
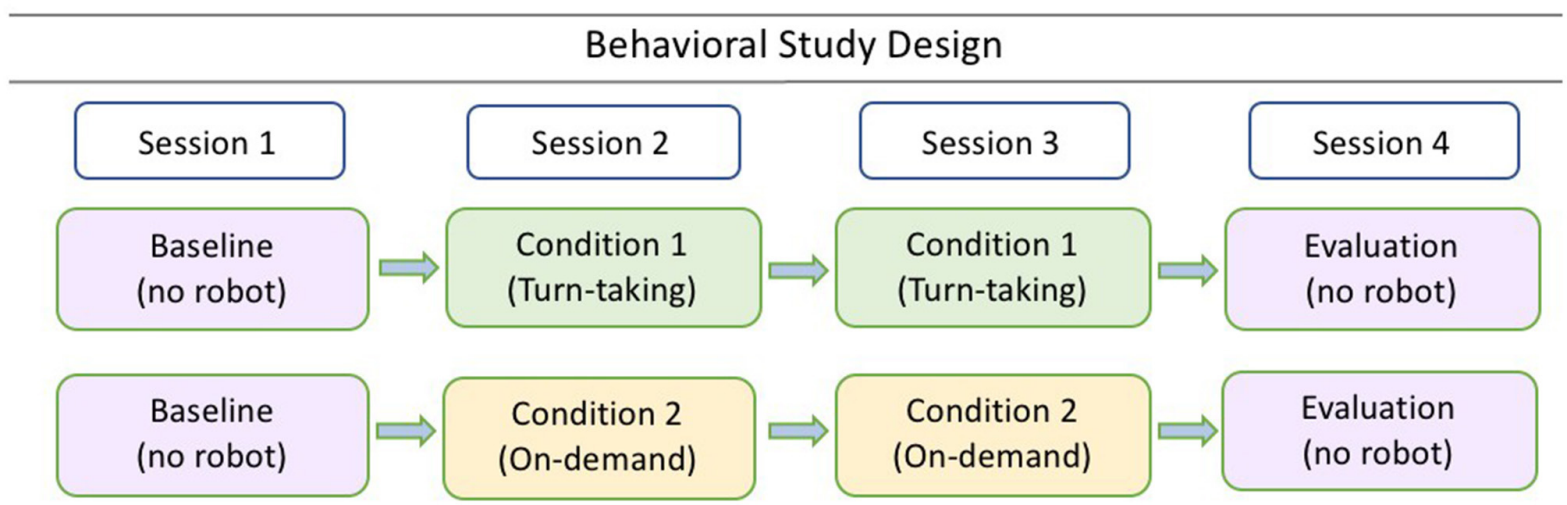
Behavioral study design with four repeated sessions per condition.

For the execution of this study, the participants are administered the Tower of Hanoi (ToH) task (Hinz et al., 2013) which is characterized by incremental task complexity. They are individually tested in 4 sessions of approximately 10 minutes each (Figure 1). First, a *Baseline Session* (BL) is conducted without any robot intervention. This session is followed by two *Intervention Sessions* during which the robot intervenes by suggesting the next optimal movement in the ToH after a child's movement; finally, a fourth session is conducted as *Evaluation Session* (EV) without the presence of the robot. An experimenter is present during the sessions who follows a predefined protocol (see complementary material); however, her role is restricted to provide initial instructions only. In order for us to eliminate any possible procedural bias during the experimental session, the experimenter is sitting inside the room avoiding exhibiting any attention to the child's interaction with the robot and the task performance.

### 2.4. Participants

Participants includes *N* = 20 (13 boys) typically developing children with an average age of *m* = 7.7, *SD* = 1.4). Of those children, four are 6 years old (yo), seven 7 yo, three 8 yo, three 9 yo, two 10yo, and one 11yo. Given the developmental nature of the current study with *N* = 4 repeated sessions per child, the sample size is identified to 20 children. The decision for the specific sample size is supported by the fact that this microgenetic exploratory study requires a different approach than experimental studies with detailed analysis of children's behavior development and typically is performed with smaller sample sizes than the ones in experimental research. Lastly, the selected sample size is in accordance to similar previous long-term child-robot interaction studies e.g., *N* = 19 children (Leyzberg et al., 2018) and *N* = 14 children (Kory-Westlund and Breazeal, 2019). One child from Cond2 (voluntary interaction) did not complete the evaluation session and six children from Cond2 did not complete the baseline session; however, we decided to take into consideration their performances during the intervention sessions, since this would provide further input to our observations of the developmental process. The children in this age differ in the degree of intrinsic motivation for task engagement and cognitive abilities. This variability in the sample provides further opportunities for the identification of various developmental patterns during problem-solving activities, which is one of the purposes of this study.

Our analysis includes 72 sessions with 113 tasks from 20 children. Of those, 10 children (4 females and 6 males) (*M* = 7.9, *SD* = 1.44) are assigned to Cond1 and 10 (3 females, 7 males) (*M* = 7.6, *SD* = 1.57) to Cond2. None of the children has any previous experience with the chosen task and any robotic platform; to eliminate any possible novelty effect, we conduct an introductory session with all participant children during which we perform the manipulation check to examine the legibility of robot behaviors.

This research study was approved by the committee on the Use of Humans as Experimental Subjects of the Joint Research Center of the European Commission; parental informed consent was obtained for all participants and all children assented to participate.

### 2.5. Materials

#### 2.5.1. The Robot

**Figure 2:**
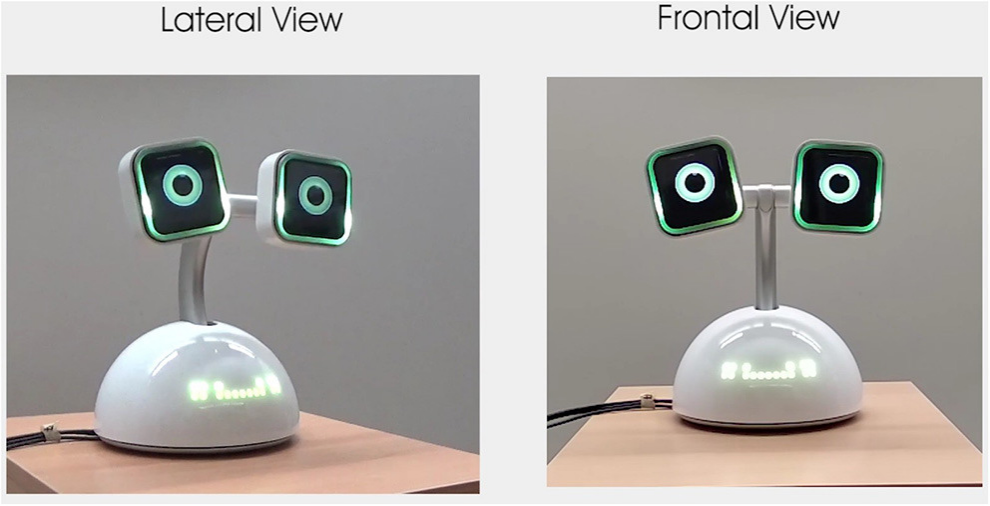
The Haru Robot with mouth LEDs in green and normal eyes on LCDs.

The robot platform considered in the study is Haru (Gomez et al., 2018), a tabletop robot for research on social robotics (Figure 2). It presents different modalities for actuation. It can move in 5 degrees of freedom (base, neck, eyes tilt, eyes roll, eyes stroke). The eyes have LCD screens that can play any video. LEDs are present in the mouth and eyebrows of the robot, and it incorporates a set of speakers and microphones. Besides the microphones, the robot uses an external Kinect camera for perception.

**Figure 3:**
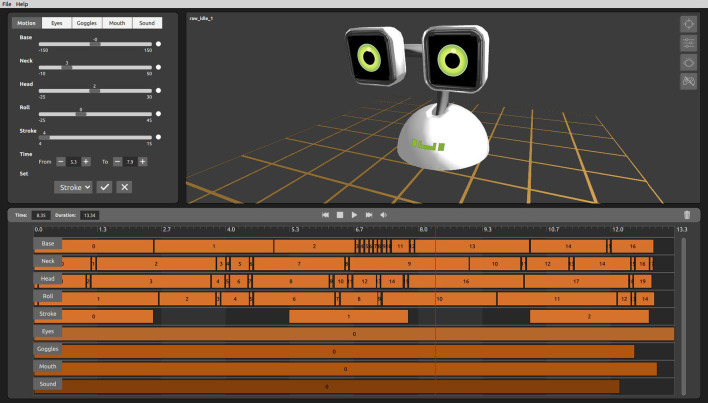
A routine generator application is used to combine the different actuators of the robot (motion of 5 degrees of freedom, eye videos, sound, etc.) to define the open-loop behaviors.

The different actuators can be controlled in real time. Also, all these elements can be combined to generate open-loop robot macro-actions mixing movement, eye motion or sounds (see Figure 3). These macro-actions are denoted behaviors in this study.

The robot is tele-operated from a control station making use of the Wizard of Oz (WoZ) technique. In the field of human-robot interaction, the WoZ technique is commonly used when the focus of the research is on the interaction design as a step before the development of the autonomous system (Steinfeld et al., 2009; Hoffman, 2016). The control station receives the images from the Kinect camera, and can be used to control directly the different actuators of the robot. Furthermore, the station allows activating pre-designed macro-actions (behaviors). For our study, we designed a set of minimally social behaviors combining the different actuators, as described below in section 2.7. This station is used in the study by the Wizard of Oz (WoZ) to control the robot, mainly by activating the corresponding behaviors adequately.

#### 2.5.2. Apparatus: The Tower of Hanoi

**Figure 4:**
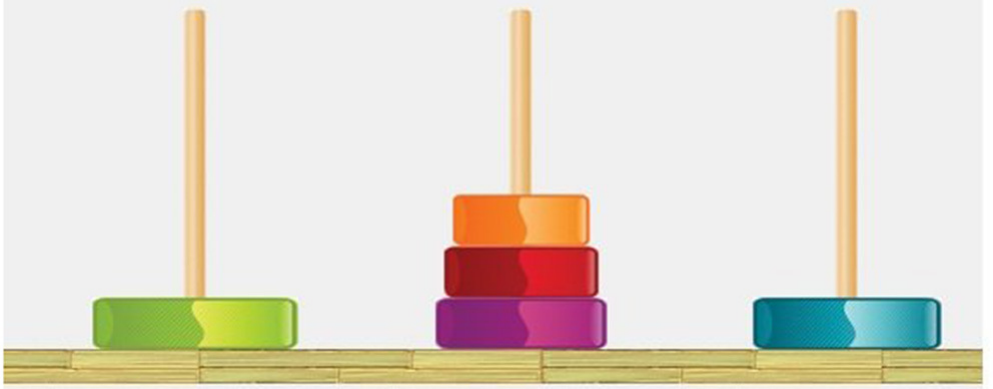
The Tower of Hanoi task.

The task considered is the Tower of Hanoi (ToH), which has a rich history in cognitive science as a problem-solving task (Simon, 1975). It involves three vertical pegs and a fixed number of colored disks with graduated sizes that fit on the pegs. At the outset, all the disks are pyramidally arranged on one of the pegs with the largest disk on the bottom (Figure 4). It requires the arrangement of disks from an initial starting point to a specified end point in the minimum number of moves, allowing the move of one disk at a time and never stacking a larger disk on a smaller one. Any number of disks may be used; the minimum number of moves for a solution is 2^*d*^ − 1, where *d* is the number of disks.

The ToH task has been used to measure children's planning abilities as well as inhibitory control; for the optimal solution, it requires the use of goal management, in which participants involve inhibition of impulsive moves that bring the child superficially closer to the goal, but are unhelpful for the longer-term solution. However, Miyake et al. (2000) note that participants may use simpler *perceptual* strategies making successive moves that lead to the display looking more like the desired end state.

The solution of the ToH requires that the child sets necessary subgoals which gradually lead to the solution of the task.

### 2.6. Settings and Procedure

**Figure 5:**
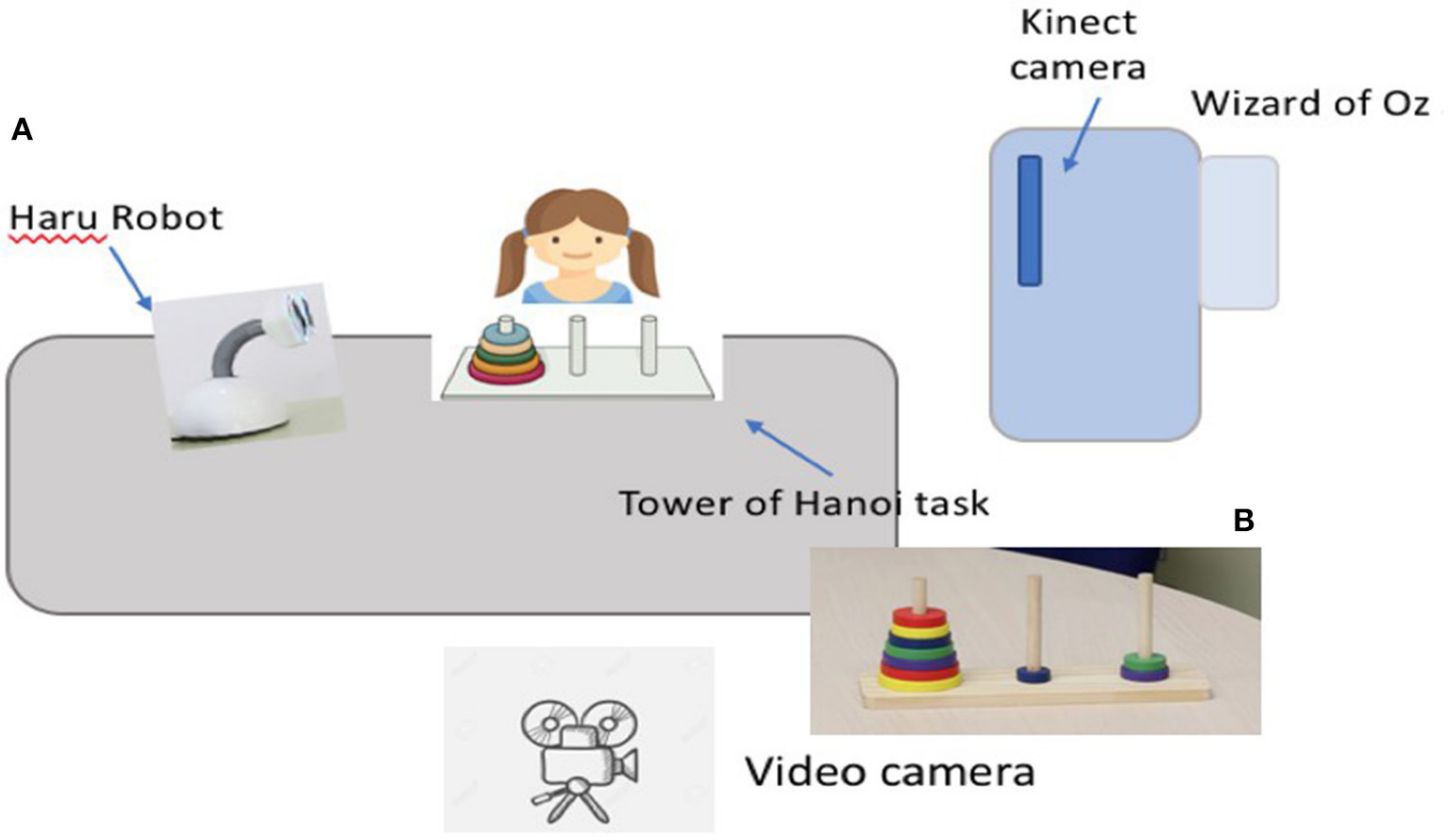
The setting of the study. The children play at a table where the robot **(A)** and the ToH instrument **(B)** are deployed. The Wizard of Oz sits in a hidden location with the teleoperation computer, and it is supported by a Kinect camera. An additional video camera is used to record data.

The study has been conducted in a primary school during a summer campus in Spain. A classroom is especially arranged for the setting of the study (see Figure 5). In the setting, a table is placed on which the physical instrument of the ToH (see section 2.5.2) and the Haru robot (see section 2.5.1) are deployed, and where the children play the game.

The teleoperation station for the Wizard of Oz is placed at a corner of the classroom, hidden from the children participating in the study. In order for us to minimize any deception effect because of the teleoperation of the robot, in the beginning of the study, we inform the children about the manner the robot is programmed and teleoperated. However, we have the teleoperation station hidden from the children in order to avoid any distraction. A Kinect camera is located on a vantage point. The video from this camera is fed to the WoZ to facilitate the teleoperation of the robot and the status of the game. Finally, a video camera for data collection is also placed in a reasonable distance from the child to eliminate any possible distraction.

Each individual child participated in four sessions over 1 week. Before the sessions all the children participated to a familiarization session (see section 2.7.3) and they were given to complete the manipulation check (see section 2.7.2). Each of the four sessions of the study was 10–15 min long. In the first session (baseline), the experimenter welcomed the child and asked for his/her assent to participate to the study; she then introduced the Tower of Hanoi rules and let the child solve the game alone. Each time the child completed a task the experimenter was asking whether the child would want to repeat the same task or continue with a more challenging one gradually increasing the number of the disks. In the end of the session the experimenter asked the child whether he/she wanted to continue the next day. In the second session the experimenter introduced the robot and explained the role of the robot depending on the condition. After the end of the four sessions, the children were interviewed about their perceptions of the robot's social competence. However, those results are not reported in this study.

Regarding the procedure followed by the robot, the WoZ is in charge of activating the corresponding robot behaviors (described in the next section) in a timely manner. Furthermore, the WoZ estimates the state of the game to indicate the robot suggestion during its turn in Cond1, or to provide help when asked in Cond2 (the next movement is provided by the robot through its LEDs, indicating the color of the disk and the peg to be moved to). The WoZ also determines when a child is asking for help in Cond2 either verbally or through a button.

### 2.7. Design of Robot Behaviors

#### 2.7.1. Overview of Robot Behaviors

We constructed a simple behavioral repertoire for the robot that supported the illusion of agency focusing mainly on goal-directed actions and avoiding expressive actions. The robot behavior design was considered as a combination of (i) the type of the behavior and (ii) the timing of the behavior performance. Regarding the type of behaviors, for the purposes of the current study we designed a set of sonic and gestural non-verbal robot behaviors. In order for us to minimize any possible effect on the expectations that verbal interaction might elicit and on children's intention for initiation of the interaction with the robot, we included only non-verbal behaviors.

More specifically, for the design of different types of behaviors, we used body and eye movements as gestural robot behaviors, sounds, and LED lights to design eight robot behaviors (see Table 1). These behaviors were all functional, targeting mainly child's cognitive engagement with the task - with the exception of the starting and ending greetings. The set of behaviors included two types of greetings, two types of providing feedback, three types of task-related behaviors, and one type for indicating that the robot was processing information. In order for us to eliminate any effect which could be related to the type of behaviors of the robot, we kept to the minimum variation of the types of robot behavior. The design of all behaviors was based on previous literature from the field of HRI, design and psychology and were based on minimalistic principles (Saulnier et al., 2011; Cha et al., 2016).

**Table 1:** Robot behavior repertoire.

**Robot intention**	**Robot executed behavior**
Greeting hello to the kid	The robot rotates the basis (45° right, 90° left, 90° right, 45° left) it stands still, it rotates the eyes (45° right, 90° left, 90° right, 45° left), it performs sound
Indicating the start of the game	The robot performs a dancing movement and looks at the task
Indicating child's turn	Robot looks at the task and looks at the child
Indicating processing current information	Looks at the task, sequence of different colors LED around the eyes moving toward outside
Suggesting the next movement	Instant suggestion of the color of the Disk and the number of Peg (visual projection on the screen of the eyes and the body
Informing that the movement was optimal	Looks at the task—looks at the child and green happy (once)
Informing that the movement was suboptimal	Repeatedly looks at the task looks at the child (x2)- wiggles NO
Greeting goodbye to the kid	Repeated rotation of the eyes (45° right, 90° left, 90° right, 45° left, LED white softer, fading sound

Regarding the timing of intervention, it only related to the cognitive task-related suggestion of the next optimal movement. The robot could give suggestions either in a turn-taking setting (Cond1) or in a setting of voluntary interaction (on-demand, Cond2). For the turn-taking setting, the robot would intervene in turns with the child by providing feedback on the previous movement made by the child, followed by a suggestion for the next task-related optimal movement. For the voluntary interaction setting, the robot would intervene only in the case in which a child would ask for help. The child could ask for help either verbally or with the use of a help button.

#### 2.7.2. Manipulation Check

We conducted a manipulation check for the confirmation of the legibility of the robot's behavior. It was designed as a single group-session with all participant children. During the session, the robot performed the designed behaviors and we asked the children to indicate their perceived robot intention in the form of a written task. The experimenter and the research group facilitated the session by introducing the robot, explaining the purpose of the session and guiding the performance of the behaviors and the gathering of children indications. In total *N* = 20 children participated to the manipulation check. We included four action-directed behaviors (Greeting hello to the kid, Informing that the movement was optimal, Informing that the movement was suboptimal, Greeting goodbye to the kid); finally we examined two additional expressive behaviors (happy and sad) in order to justify children's understanding of the current task. The results of the manipulation check show that 71, 43% of children's answers were accurate regarding the legibility of robot's behaviors. More specifically the behavior with the higher percentage of legibility was the Sad behavior with 100% correct answers. These results justified that the children understood the current task. This was followed by the “Informing that the movement was optimal” behavior with 91, 67% correct answers. The least legible behavior was the “Greeting hello to the kid” behavior with 50%, which was confused with the “happy” expressive behavior. However, given that the manipulation check was performed in a de-contextualized manner we expected that there might be a confusion between the goal-directed and the expressive actions which are not mutually excluded.

#### 2.7.3. Familiarization Phase

For the elimination of any novelty effect on children's behavior, following the manipulation check, we allowed the children to informally interact with the robot. This informal activity lasted 10 min and was designed to be unstructured. Each child was free to interact with the robot at his or her willingness and the researchers did not impose any kind of interaction. All the children remained into the classroom for the informal activity.

## 3. Metrics and Analysis

Audio and video recordings of the study sessions were recorded with two cameras for later transcription and off-line analysis. A first iteration of the recorded sessions observation as well as the initial hypotheses of the study lead us to the development of the annotation scheme. As it was expected, since the robot did not exhibit any verbal behavior, the child-robot verbal interaction was minimum. The only case the children were addressing verbally to the robot was during the Condition 2 of the voluntary interaction when they asked for help verbally—in addition to the option of asking for help with the use of a button. For this reason, the verbal interaction data reported in this paper only includes child's verbal behavior of “asking for help.”

The recorded video was used to transcribe children's task-related behavior as well as social interaction with the robot and verbatim. However, for the purposes of the research question addressed in this paper, we only report the task-related behavior. Participants' behaviors were manually annotated off-line by an instructed annotator. Because of the objective nature of our coding scheme (disk movements, asking for help Cohen's K and breaking the rules), which did not require any subjective interpretation of children's behavior, we run a set of sessions during which the two coders annotated the same extracts. During those sessions, any minor disagreement was discussed with the first author of the paper, which resulted in a consensus of the coding.

We annotated in total 72 individual sessions. In each one, more than one task could be included, depending on the duration of child's task performance. This resulted in the annotation of 113 tasks from 3 to 7 disks of the Tower of Hanoi. The annotation scheme included (i) the occurrence of task-related actions (disk movement); (ii) the use of help button or child's verbal asking the robot for help; and (iii) the instances of breaking the rules of the game. In addition, we chose *N* = 4 case studies (see section 4.3), which correspond to 16 sessions (BL, Interventions and EV) to annotate the characterization of the child's task-related action (optimal or suboptimal, see below).

We observed that because of the canonical robot intervention in the sessions of the turn-taking condition, the sessions in Cond1 lasted longer than the ones in Cond2. For this reason, we normalized the sessions duration taking into consideration the optimal number of movements per task. However, we did not consider the sessions duration in our data analysis because robot's canonical interventions in Cond1 (turn-taking) and the on-demand intervention in Cond2 would create an imbalanced comparison between the two conditions. For this reason, our data analysis only focused on children's task-related actions.

Using the off-line video annotation tool ELAN^1^, we manually annotated the data according to the annotation scheme.

### 3.1. Task Performance

To measure the performance of a given task, we annotate individual movements and compute the difference between the number of movements *L* and the optimal number of movements *O*_*d*_ for the number of disks *d* of the task. In order to compare this metric for tasks with different number of disks, we then normalize this value by the optimal number of moves in task as follows:

(1) $$\begin{array}{l} K=\frac{\Delta L}{O_{d}}=\frac{L-O_{d}}{O_{d}} \end{array}$$

where *d* varies from 3 to 7 disks and $O_{d}=2^{d}-1$^2^. Please note that, as defined, higher values of the metric *K* indicate lower performance.

Since the child was free to choose whether after the completion of one task she/he would continue to the next task by increasing the number of disks or not, we considered for our analysis the total number of tasks per disk, which might exceed the number of participants.

In the following analyses, we ran a Kolmogorov–Smirnov test to check the normality of the data. Based on the Komogorov-Smirnov result, we used non-parametric Wilcoxon test for paired samples to check the difference in children's problem-solving performance (learning) by comparing the results of the baseline and the evaluation session, and Mann–Whitney's *U*-test for non-paired samples to compare the results of the evaluation session between the two conditions.

### 3.2. Voluntary Interaction

In Cond2, we designed a voluntary HRI setting with an “on demand” robot intervention as an indicator of child's intrinsic motivation for problem-solving. We annotated and counted the instances in which the child was explicitly asking the robot for help either verbally or using a help-button. We normalized the number of instances with respect to the number of optimal moves per disk *O*_*d*_ to obtain a measure for help *H*. This normalization was done to obtain comparable values for different tasks.

### 3.3. Task Improvement

As being a developmental study, we also analyse the development of child's task performance *K* along the different sessions. Thus, we analyzed the improvement between (i) the first and the last task of the baseline; and (ii) the last task of each session and the first task of the following session, by computing the difference of normalized extra moves metric *K* in both cases.

### 3.4. Developmental Process

**Figure 6:**
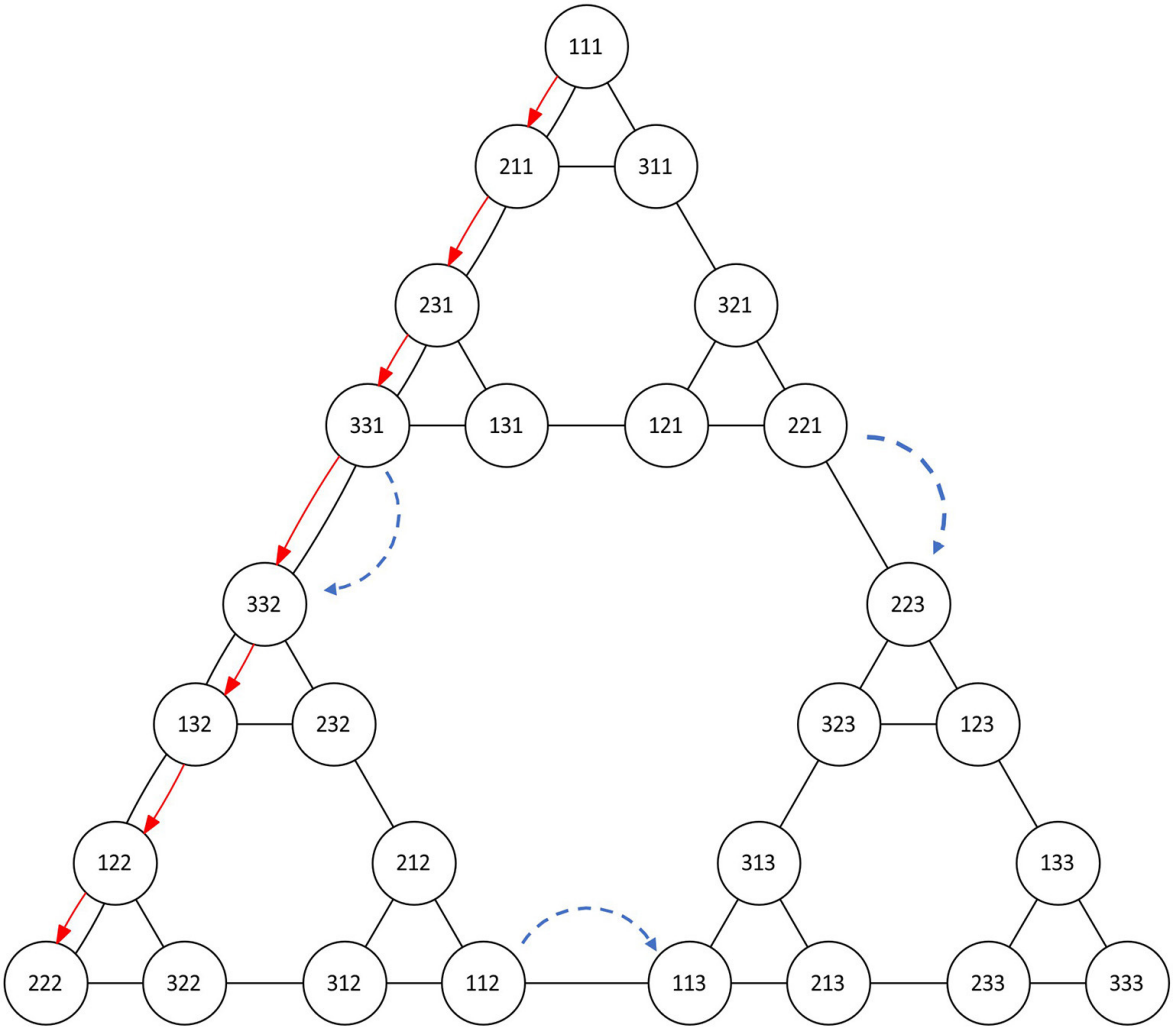
Graph representation of the ToH game for *d* = 3 disks. Each node represents one disposition of the disks. Red color represents the optimal path between the initial disposition and one solution. In blue dashed, auxiliary movements (movements between sub-graphs leading toward the solution).

The Tower of Hanoi game can be represented as a graph (the Hanoi graph) (Knoblock, 1990; Hinz et al., 2013), as illustrated in Figure 6, in which each node represents a legal disposition of the disks on the pegs (for instance, for the 3-disk case, the node 112 represents the smallest disk in the 2nd peg, and the other disks in the 1st peg) and edges represent valid movements between nodes.

For *d* disks, there are 3^*d*^ nodes. Under a certain positioning of the nodes, the graphs resemble the Sierpinski gasket (Hinz et al., 2013). We used this model to manually annotate each sequence of movements and relate it to the optimal path, understood as the solution with the minimum amount of moves *O*_*d*_. Due to the high cost of manual annotation, we carried out these annotations for all tasks but only for subgoals with a maximum of 5-disks, i.e., *d* ≤ 5.

We annotated the type of movements as follows: (i) Optimal/sub-optimal: optimal moves refer to the moves which are on the optimal path toward the solution or function as recovery actions (sub-optimal) toward the optimal path; and (ii) Auxiliary: Auxiliary movements refer to those that use a third peg as a scaffold for the optimal solution of the task. Within a task of a certain number of disks there are subgoals; these are instances of milestones of a subpyramid that leads to the task solution (see Figure 6).

## 4. Results

We used the above-mentioned metrics to address the research questions and the corresponding hypotheses as follows.

### 4.1. Task Performance in Turn-Taking and Voluntary Interaction (Hypothesis 1)

We hypothesize that children in Cond2 (voluntary interaction) would be more likely to show better performance in the evaluation session than children in Cond1 (turn-taking). To explore this hypothesis, we consider (i) children's task performance and (ii) task improvement over the four sessions, with a focus on the evaluation session. We note that, during the intervention sessions, children in Cond2 had the opportunity to perform more movements than children in Cond1 since in Cond1 the robot provided canonical intervention in a turn-taking setting.

#### 4.1.1. Task Performance

For each task, we assess the value of *K* (normalized extra moves), as detailed in section 3.1.

Mean performance metrics are summarized in Table 2 (BL and Cond1) and Table 3 (Cond2). The average is presented in relation to the incremental task complexity (number of disks *d* with *d* = 3…7). For the baseline, we integrate the performance of participants in both conditions as there is no difference in the setting.

**Table 2:** Task performance metrics Δ*L* and *K* in Baseline (left column) and in Cond1 (turn-taking) per session and per number of disks.

**d**	**Baseline**			**Cond1-Intervention**			**Cond1-Evaluation**		
	**t**	**ΔL**	**K Mean (SD)**	**t**	**Δ L**	**K Mean (SD)**	**t**	**ΔL**	**K Mean (SD)**
3	18	2.78	0.40 (1.24)	1	3	0.43 (0)	–	–	–
4	16	15.38	1.03 (0.79)	6	4.67	0.31 (0.17)	–	–	–
5	5	36.40	1.17 (0.99)	13	4.69	0.15 (0.10)	2	109	3.52 (2)
6	–	–	–	14	7.28	0.12 (0.11)	4	112	1.78 (0.33)
7	–	–	–	–	–	–	5	201.6	1.59 (0.45)

*t represents the number of considered tasks*.

**Table 3:** Task performance metrics Δ*L* and *K* in Cond2 (voluntary interaction) per session and per number of disks.

**d**	**Cond2-Intervention**			**Cond2-Evaluation**		
	**t**	**ΔL**	**K Mean (SD)**	**t**	**Δ L**	**K Mean (SD)**
4	7	6.86	0.45 (0.17)	1	9	0.6 (0)
5	12	20.5	0.66 (0.10)	2	31	1 (0.15)
6	10	74.5	1.18 (0.11)	2	161	2.55 (1.41)
7	–	–	–	4	–	1.15 (0.38)

*t represents the number of considered tasks*.

As expected, in the baseline, we observe an increase in the normalized extra moves *K* for the task with increased difficulty (more disks), ranging from 0.40 (*d* = 3) to 1.17 (*d* = 5). In the intervention session of Cond1 the normalized number of extra movements seem to not be associated with the increased difficulty of the task ranging from 0.12 (*d* = 6) to 0.43 (*d* = 3) with relatively small deviation from the optimal solution path during the robot's canonical intervention. However, in the intervention session of Cond2, the extra movements range was between 0.45 (*d* = 4) to 1.18 (*d* = 6) which is a larger deviation from the optimal path than in Cond1. As expected, the task performance is linked to the difficulty of the task in terms of number of disks.

Interestingly, in the evaluation session in Cond1, task performance *K* ranges from 1.59 (*d* = 7) to 3.51 (*d* = 5), and the deviation from the optimal solution is higher in the first task of the session than in later stages with increased difficulty. However, in the evaluation session of Cond2, *K* is smaller than for Cond1, ranging from 0.6 to 2.55 which indicates a smoother transition from the intervention to the evaluation session in the voluntary interaction case. It should be noted that in many cases the Standard Deviation of the selected metrics is relatively large, which indicates a large distribution, most likely because of the small sample size.

#### 4.1.2. Task Improvement

**Figure 7:**
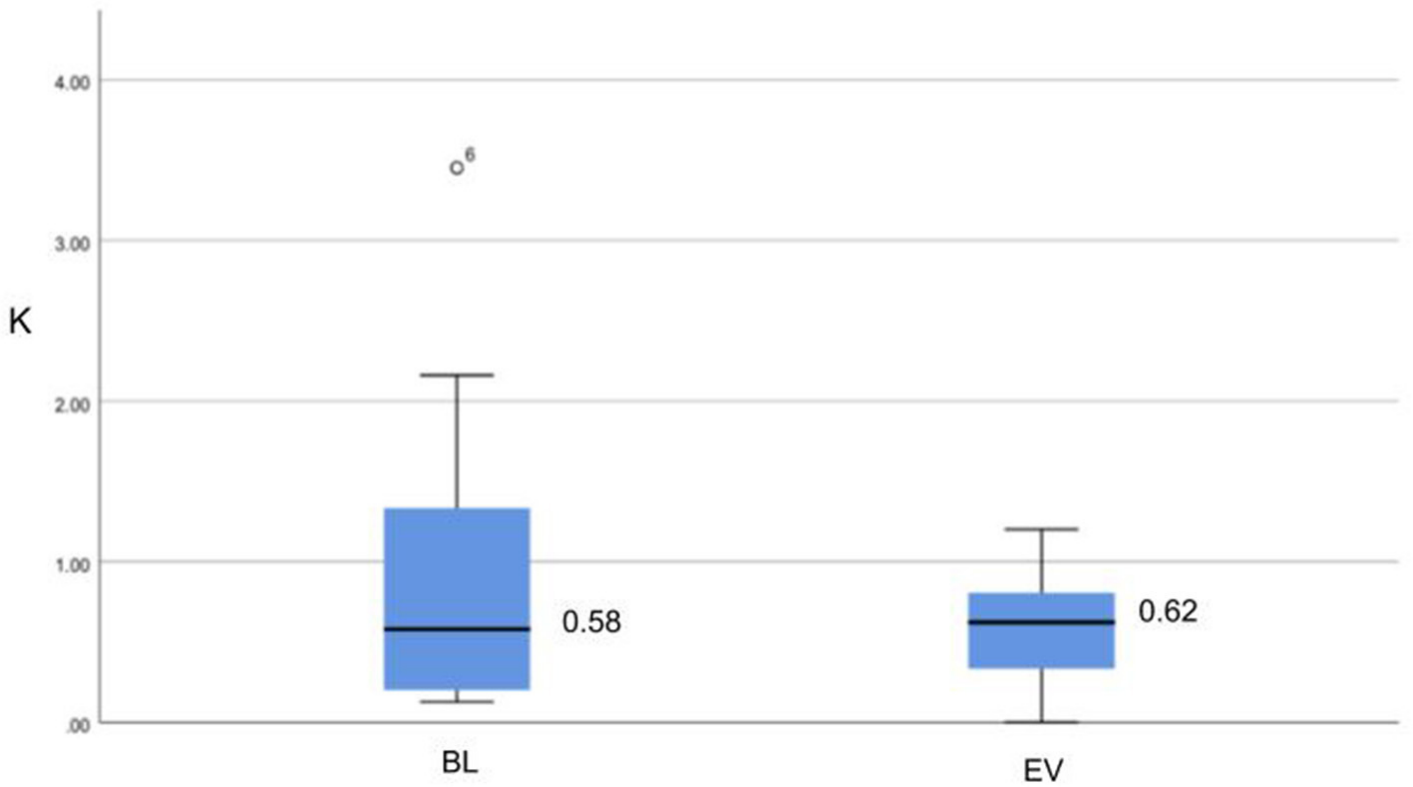
Distribution of task performance *K* for BL and EV sessions in both conditions. Median values are displayed.

Figure 7 shows the distribution of task performance for BL and EV sessions considering both conditions. We observe that the median value is higher in evaluation than in baseline, indicating an overall learning effect. In addition, we computed individual differences in task performance Δ*K* between EV and BL session. It should be noted that, since we measure the difference in normalized extra movements, a negative difference indicates task improvement. Our descriptive results show an average Δ*K* = −0.326 (or 35% if instead of difference we compute the percentage decrease), which also reflects a better average performance in the EV session. However, the results from the Wilcoxon test between BL and EV value distributions showed no statistical significance (*p* = 0.286, *a* = 0.05). As discussed below, due to the increasing difficulty of the task in the EV session, our findings might indicate a learning tendency.

**Figure 8:**
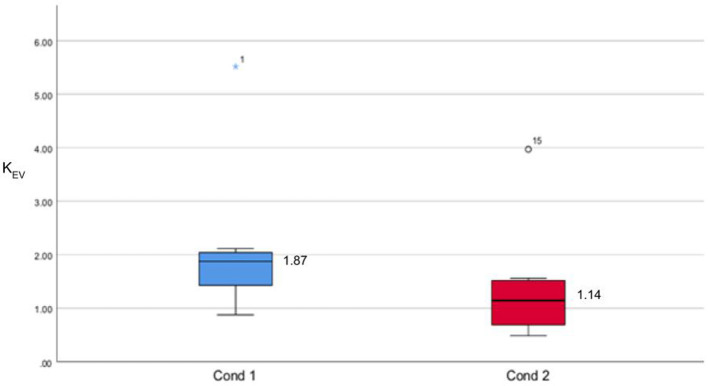
Statistical distribution of task performance *K* in evaluation sessions of both conditions. Median values are displayed.

In addition, we performed a Mann–Whiteney's *U*-test to check the statistical difference between EV sessions in Cond1 and Cond2. Statistical distributions are illustrated in Figure 8. Results show significance of *p* = 0.038 (*a* = 0.05). The interval of confidence for the difference between Cond1 and Cond2 is between 0.020 and 1.237, which means that the performance is significantly higher in Cond2 than Cond1.

**Figure 9:**
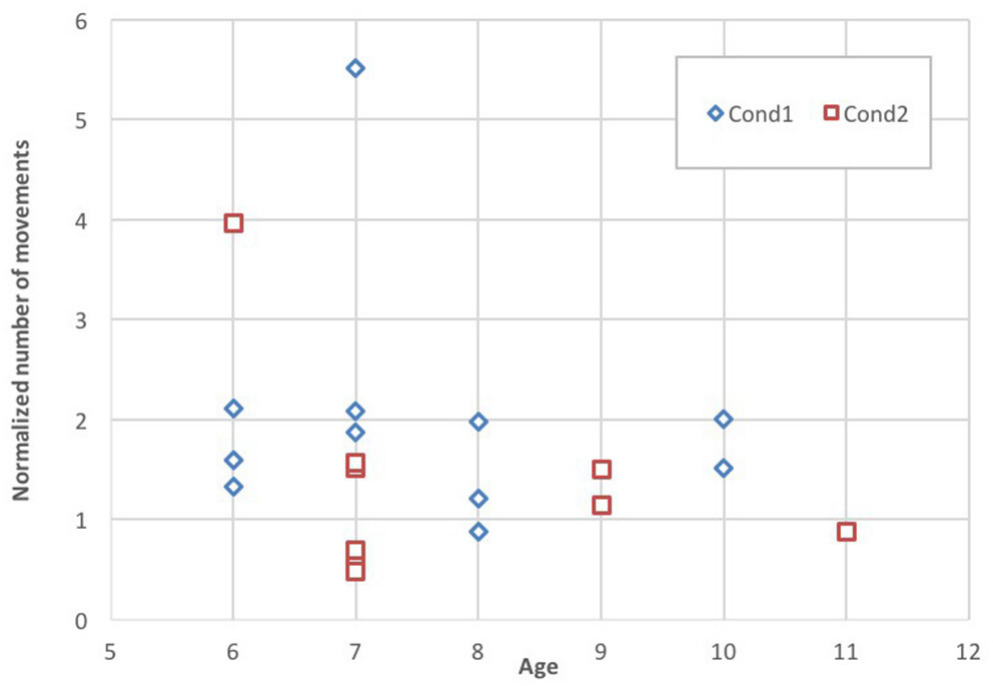
Individual task performance *K* versus age in the evaluation session for Cond1 and Cond2.

Lastly, we looked at the possible association of those results with the age of the children (Figure 9). Our results show that in the evaluation session of Cond1 most of the children of any age perform larger numbers of movements than in Cond2.

### 4.2. Children's Tendency for Self-Initiated Robot Interaction (Hypothesis 2)

We hypothesize that in the voluntary interaction the participant children who faced more difficulties in solving the task (e.g., younger children) were more keen to ask the robot for help. We expected this because child's learning often occurs in collaborative settings with the scaffolding by others (Vygotsky, 1978). To explore this hypothesis, we considered (i) the number of the instances the individual child asked for help (asking for help) in relation to the task performance (extra movements) and (ii) the age of the child. Because of the incremental nature of the task, there were more opportunities for children to ask for help; for this reason, we normalized the scores in order to be comparable.

We assess child's voluntary interaction in relation to the task performance during Cond2 as well as the task improvement in the evaluation session.

**Figure 10:**
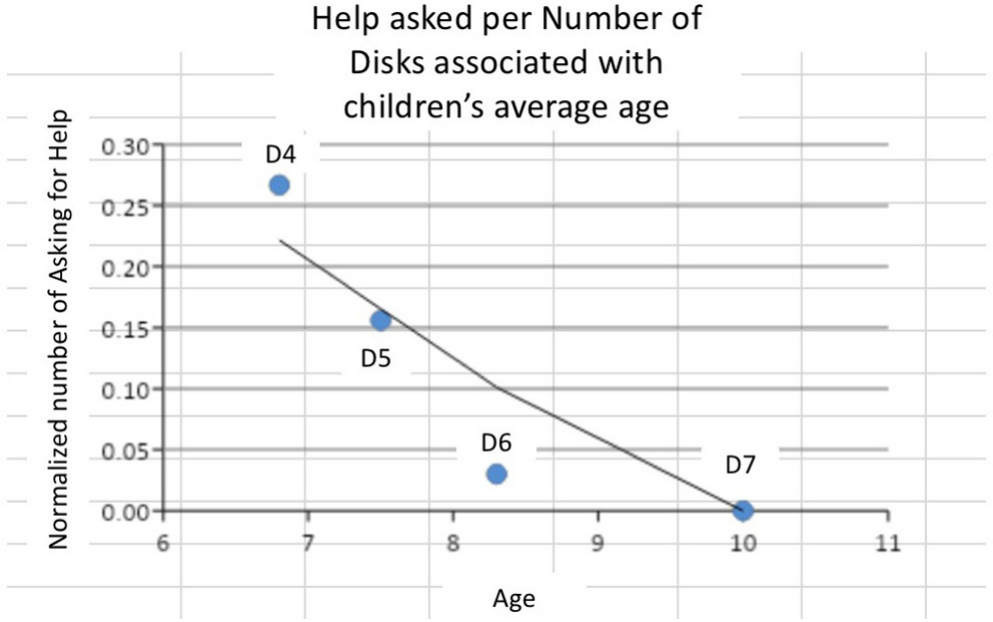
Average *H* metric of child's asking for help in Cond2 vs. child's age, presented according to number of disks.

During the robot intervention in voluntary interaction, we observed a trend for more instances of asking for help in less demanding tasks by younger children (Figure 10). More specifically, for *d* = 4 tasks, children of average age 6.8 years exhibit *H* = 0.26 asking for help behavior. For *d* = 5 tasks, children of average age 7.5 years exhibit *H* = 0.16 asking for help behavior. For D6, children of average age 8.3 asked for help *H* = 0.03 instances and for *d* = 7 the only child that managed to perform the task with 7 disks in the intervention session was a 10 year-old who didn't ask for help at all.

**Figure 11:**
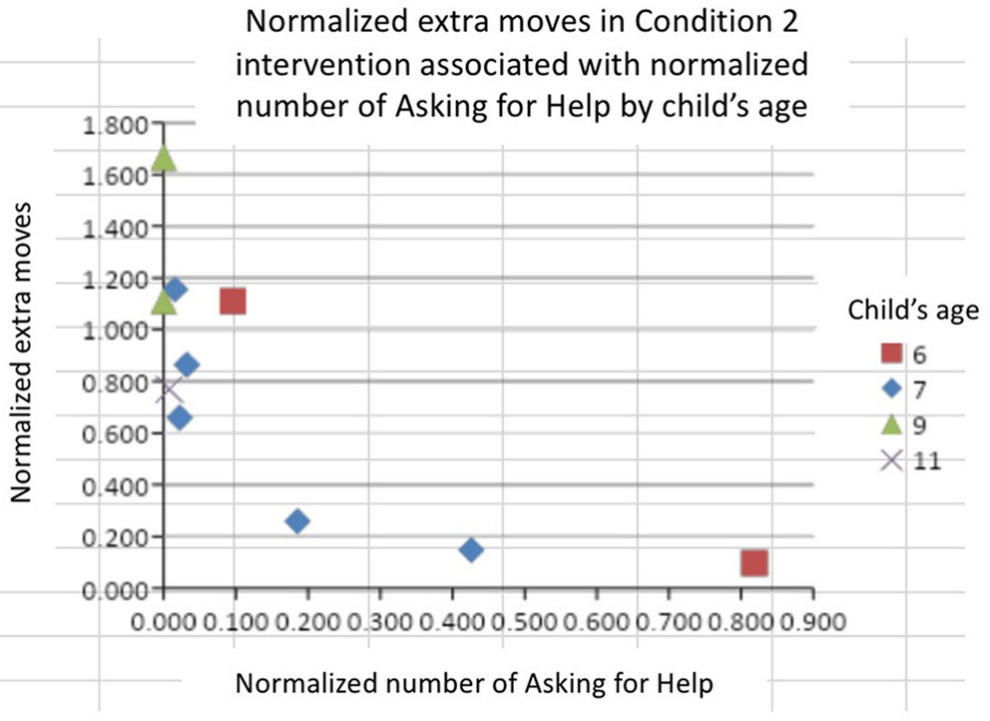
Representation of *K* vs. *H* to represent normalized number of movements with respect to help by the robot.

In addition, we considered the score of extra movements for these children. As shown in Figure 11 in total 9 out of 10 children asked for help during the robot intervention. Of those, 6 children showed increased number of extra movements ranging from *K* = 1.11 to *K* = 1.66 with low number of instances of asking for help (normalized range from *H* = 0.00 to *H* = 0.096). On the contrary, three children exhibit increased number of instances of asking for help, ranging from *H* = 0.18 to *H* = 0.82, with decreased number of extra movements, ranging from *K* = 0.25 to 0.097.

### 4.3. A Single Case-Study, Pattern Emergence, and Inter-individual Differences (Hypothesis 3)

To gain a more refined understanding of the problem-solving process and to identify possible patterns in action sequences, we map the developmental trajectories of the task solution for *N* = 4 selected children. The selection of the specific case studies was based on their representiveness in terms of the solution path that the children followed during the sessions. In this section we analyse one single case study and we selectively juxtapose instances from the remaining three case studies.

For our analysis, we assessed all movements as optimal or suboptimal and mapped it to the visual representation of the ToH solution presented above (see Figure 6). We used the visualization to map the sequence of child's task-related actions and to define possible emerging patterns.

#### 4.3.1. Baseline Session

##### 4.3.1.1. Optimal performance

The child “Sophie,” aged 8 years, participated in Cond1 of the study. During the baseline session, without the presence of the robot, Sophie understood the rules of the game and showed a positive stance toward the game and the activity. She started solving the task with *d* = 3 disks, without facing any difficulty. We observed that toward the end of the solution, Sophie increased the pace of her task-related actions. This has been registered as a typical behavior that was observed repeatedly in all participant children and can be explained by the cognitive theories that describe child's *perceptual* strategies making successive moves that lead to the display looking more like the desired end state (Miyake et al., 2000).

##### 4.3.1.2. Deviation

**Figure 12:**
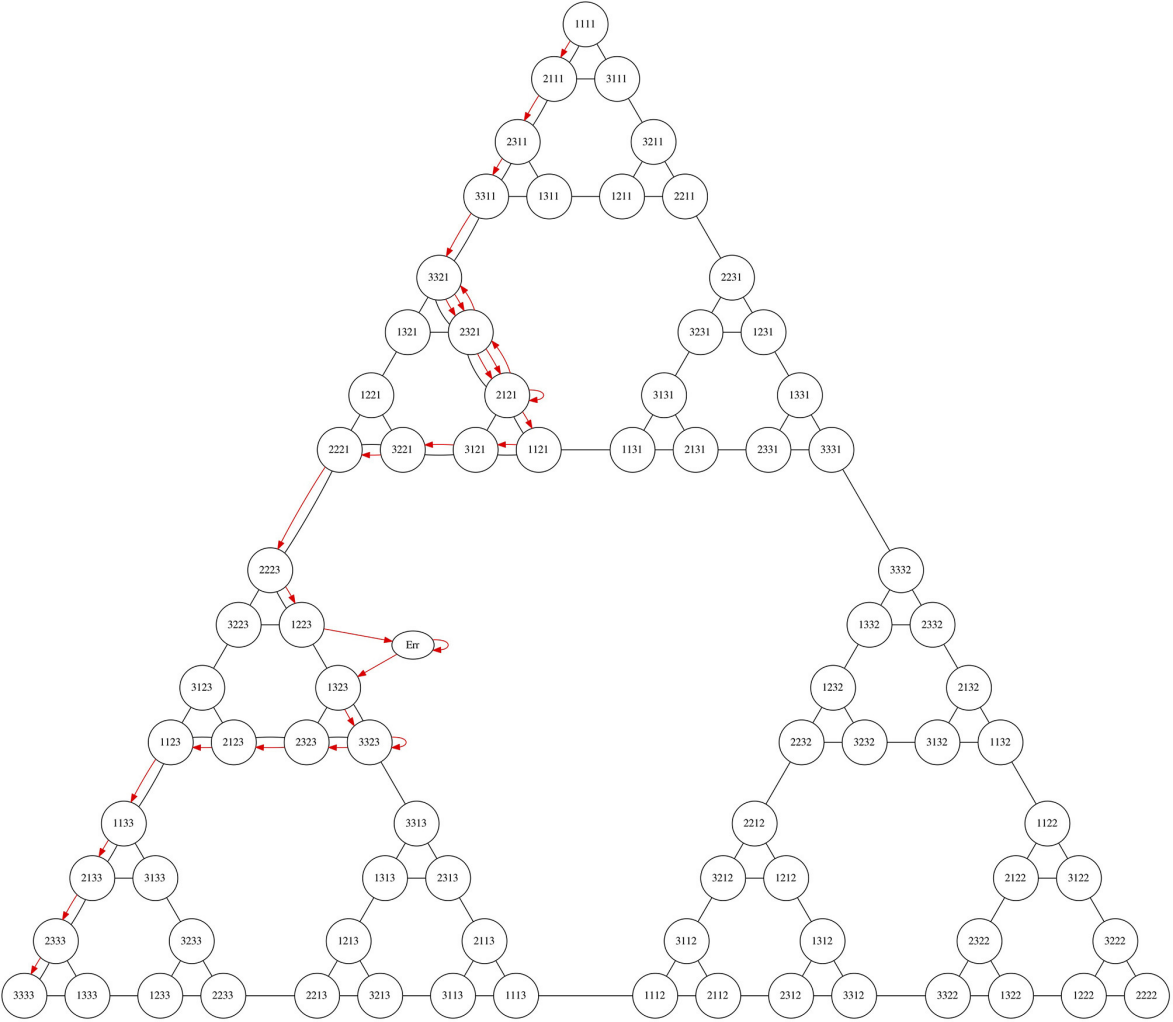
A developmental representation of individual child's problem-solving path of the baseline. The figure shows a pattern of frequent deviation from the optimal solution path.

Then, Sophie proceeded to the next task with *d* = 4 disks. While in the beginning of the task, we observed an increased pace in her actions, after the movement 4, the solution pace was diminished and, as shown in Figure 12, she started deviating from the optimal solution path. The point she started to deviate was the instance where she should perform an auxiliary movement and inhibit inappropriate move selection. This demand appears at specific points where there is a mismatch between the end goal of the problem and a current subgoal. This was a typical behavior that appeared in the Baseline session in all the four case studies we evaluated.

##### 4.3.1.3. Recovery

After four movements, Sophie understood that she was not on the optimal solution path of the task and she started performing recovering actions. We observed an increased pace of her actions during the recovery which might be explained by theories that focus on executive function of planning (e.g., Miyake et al., 2000).

##### 4.3.1.4. Inhibitory control points

The solution of the *d* = 4 disks ToH task requires from the child at least three instances of inhibitory control. At those points the child should perform an auxiliary movement in order not to deviate from the optimal solution path. However, Sophie did not make use of the auxiliary movement which resulted in a canonical deviation from the optimal solution path as appears in Figure 12.

##### 4.3.1.5. Child-initiated interaction

In condition 2, we annotated the child initiated interaction indicated by the instances of the child's asking for help as described in section 4.2. The microgenetic assessment provides further insights on the timing of child-initiated interaction. As expected, we observed that the majority of the instances appear on the nodes where the child had more than one options to perform the next movement with higher probability to deviate from the optimal path. This coincides with the auxiliary actions that indicate the child's inhibitory control.

##### 4.3.1.6. Pattern emergence of developmental sequences

The pattern which appears in Sophie's baseline for the *d* = 4 disks task appeared in all the four case studies we analyzed. In a similar way, a typical solution path in turn-taking condition consisted of optimal moves only following the diagonal axis of the triangle. In the evaluation session, the child exhibits canonical deviation from the optimal solution path with an improvement from the baseline, and a more frequent deviation from the optimal path than the one exhibited in the intervention session.

##### 4.3.1.7. Pattern emergence of temporal aspects

**Figure 13:**
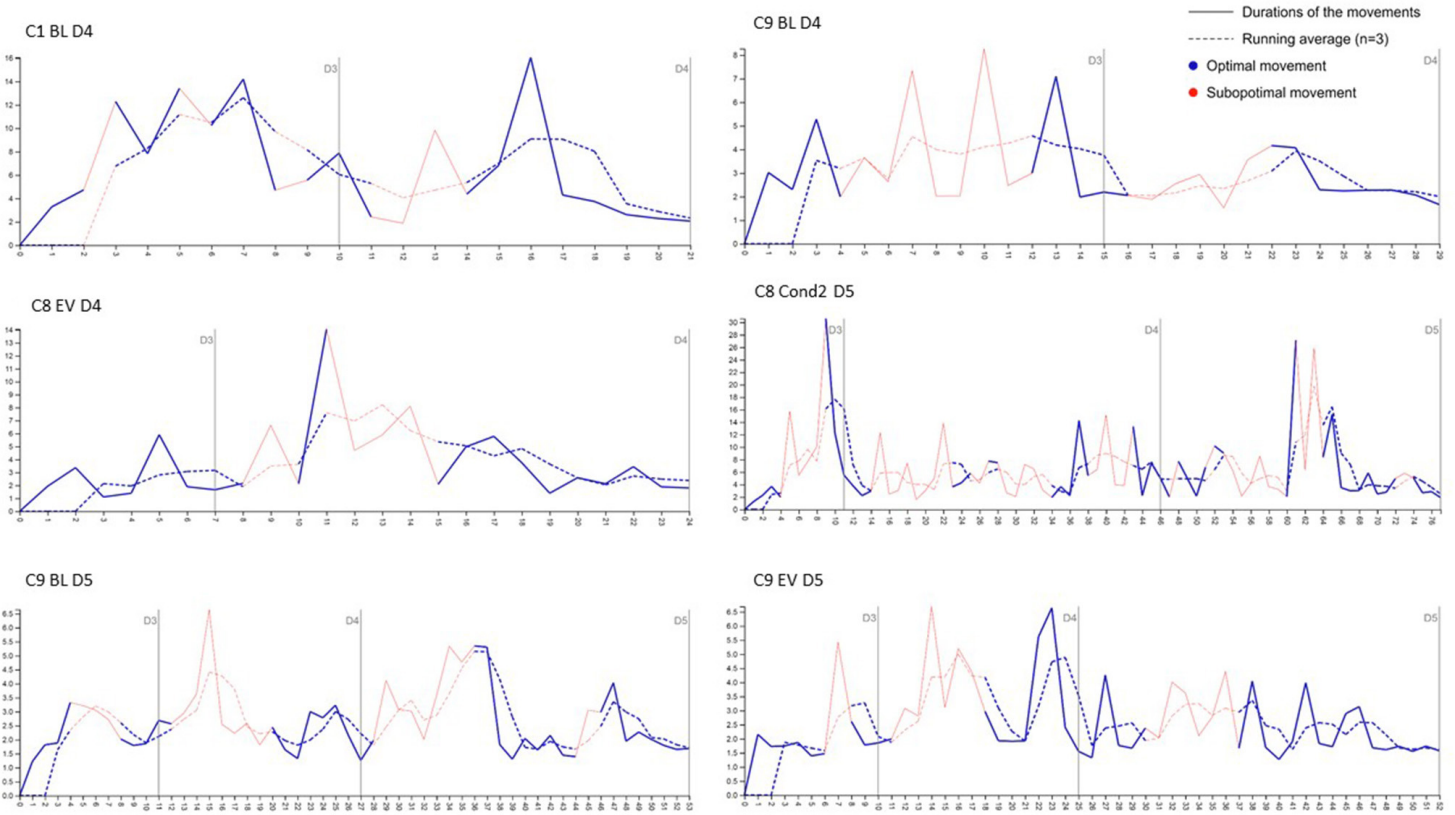
Examples of speed of optimal and suboptimal actions in association with the subgoals of the problem-solving task.

To illustrate how the problem-solving trajectory develops over time, we examine the speed of the moves throughout the task. Figure 13 shows a selected set of representative examples from the analyzed cases with the duration of each move (in seconds), in addition to a moving average of the last three movements. We observe an increase in speed (short duration) in the last movements of a subgoal for all the analyzed children. Additionally, we observe that the increase in the speed of movements toward the final solution of the tasks is associated with optimal movements, while increase in the speed of movements between subgoals of the same number of disks is associated with suboptimal movements such as exploratory actions.

#### 4.3.2. Robot Intervention Session

In the second and the third session Sophie participated to the robot intervention session in Cond1 in which Sophie was instructed to solve the ToH task together with the robot in a turn-taking setting. Sophie looked engaged with the robot and she clearly perceived all the intended behaviors of the robot. She selected to repeat the task with *d* = 4 disks which she solved in the optimal way in collaboration with the robot. As shown in Figure 12, her performance was optimal in the task with *d* = 5 disks as well when solving the task together with the robot.

While an optimal solution was typical for all children in Cond1, children in Cond2 showed different patterns of task solution, which differ depending on the frequency child asked the robot for help. In the examined case studies we observed solutions with (i) canonical deviation from the optimal solution at the points which required auxiliary movements (ii) instances of child breaking the rules of the game and (iii) solutions with extensive exploratory actions which lead to a final solution with the use of large number of extra movements.

#### 4.3.3. Evaluation

In the last session, Sophie selected to solve again the *d* = 5 disks task, without the help of the robot. While in the intervention session, Sophie solved the task together with the robot following the optimal path, in the evaluation session she regularly deviated by the optimal as shown in Figure 12. The pattern of Sophie's deviation from the optimal path in the evaluation resembles the one in the Baseline with the four disks task, in having the critical points of the use of auxiliary movements as necessary for the continuation of the optimal solution. However, Sophie's pattern of solution seems improved in the evaluation session since she achieved to use inhibitory control in four out of eight critical points. This indicates the dynamic nature of problem-solving process in incremental tasks which requires special attention to the design of robot intervention.

## 5. Discussion

In the current study, two main topics were addressed: First, we evaluated children's problem-solving task performance in a “voluntary” HRI condition in contrast with a “turn-taking” condition in a longitudinal setting. Second, we examined the developmental trajectory of the process of problem-solving via possible patterns of the sequence of actions over multiple sessions. To address the first topic we captured children's performance of the ToH task in an incremental manner looking at the role of the robot intervention on the task performance. To address the second topic, we considered children's deviation from the optimal path of the solution which allowed us to highlight the heterogeneity of children's problem-solving trajectories. Our goal was to observe children's trajectories of problem solving, and to create an HRI setting that allowed for voluntary childrobot interaction with child-initiated robot intervention. Below we discuss the main findings:

### 5.1. Exploration in Young Children's Problem-Solving

Our results indicate that participants in the “turn-taking” condition exhibit less exploratory movements than in the “on-demand” robot intervention condition. However, in challenging tasks, young children that participated in the “on-demand” robot intervention and had the possibility to perform more exploratory actions outperformed young children that participated in the “turn-taking” condition in terms of deviation of the optimal moves. Thus, our findings provide initial indications regarding young children's need for exploratory actions in problem-solving process in HRI settings and the efficacy of those actions in challenging task performance.

### 5.2. Inhibitory Strategy Emergence and Development

The cognitive strategy of inhibition has been characterized as one of the main strategies used for the optimal solution of the ToH task (Goel and Grafman, 1995). This strategy allows the child to inhibit moves directly to the goal in order to make the counter-intuitive move that leads to the optimal solution. We identify the use of inhibitory strategy in all observed optimal moves excluding the moves leading to a subgoal or the final solution of the ToH. Our design allowed us to observe that this strategy is not apparent to all young children, especially in the more challenging tasks. However, the fact that our cases increased the speed of their optimal movements only toward the reach of a subgoal indicates that the analyzed children used additional strategies for the task solution such as implicit learning. Typically, this procedural learning is observed by continuous improvement in performance over repeated administrations of the same ToH problem, as shown by our analysis of the learning effect.

### 5.3. Designing Robot Behaviors to Scaffold Child's Exploration

For the current study we used the Haru robot with minimally designed social behaviors. Since our main focus was on the type and timing of robot cognitive intervention rather than on robot's social behaviors, on purpose, we restricted the robot behaviors into cognitive interventions providing suggestions in a neutral non-verbal manner and feedback related to the task performance only. Maintaining the same behavioral principles, we designed an “on demand” robot intervention. This is one of the few studies in HRI that provide children the space to voluntarily initiate the robot intervention. Our results indicate that there is a relationship between children's intrinsic motivation for exploration and robot intervention, since in many cases the participant children did not ask for help by the robot and preferred exploration which lead to increased task performance. Additionally, the “on demand” intervention allowed for inter-individual variability to be observed, with some younger children being inclined for more exploratory actions, which might require personalized robot interventions.

However, we observed that children's deviation from the optimal solution path in the specific task comes with certain patterns. From a pedagogical perspective, these patterns can be utilized in order for designers to develop *targeted robot interventions* which allow the child to explore and experience self-initiated interactions. In addition, at targeted instances of the task, the robot intervenes in order to provide recovery in child's actions and scaffold the child's problem-solving process which would lead to better learning experience and outcomes for the child.

This paper contributes to the field of HRI as one of the few developmental studies which focuses on the process rather than only on the final outcome of child's activity and provides indications about not only the *what* but the *why* of collaborative problem solving in child-robot interaction. Further, the suggestions of voluntary interaction contributes to the current dialogue about the ways we need to develop value-centered intelligent systems. In this way the child has the freedom to initiate the interaction according to her needs.

## 6. Limitations and Future Work

Deeper insight into the trajectories of children's problem-solving will allow us to construct dedicated theoretic models for the emergence and development of children's complex strategies. In similar fashion to the work by Oudeyer and Smith (2016) on modeling curiosity development, in future work, we also intend to computationally model and simulate problem-solving processes of increasingly complex tasks. Toward this end, we intend to develop a robotic companion for dynamic assessment and support of children's tendency for exploration as one of the catalytic stages for the emergence and development of relevant cognitive strategies for problem solving. From a methodological perspective, whilst most of the current longitudinal studies with children in HRI include relatively small sample (i.e., Leyzberg et al., 2018), we aim to investigate child-robot collaboration in problem-solving tasks in a longitudinal study with a larger sample. In this way, we will be able to contribute to the dialogue regarding child development in HRI settings with generalizable results. In addition to this, we acknowledge that between the interaction design of the two conditions lie further possibilities for child-robot interaction in the context of collaborative problem-solving activities. Our plans for future work include additional possibilities for further types of interaction design.

Regarding the robotic system itself, we are currently developing a fully autonomous system for the dynamic assessment and autonomous robot intervention for the ToH task to carry out a larger scale study considering a fully autonomous interaction. This requires, from the perception part, to estimate the state of the game, the individual child problem-solving abilities and other individual characteristics. Tracking the state of the game makes it possible for the robot to automatically evaluate the task progress and thus take decisions accordingly.

Deeper data-driven analyses may further reveal characteristics and causes of child development and the transition from primitive cognitive and social actions toward more complex behaviors. As discussed before, all children did not have explicit conceptualized knowledge and strategies for problem-solving of the ToH task. So interacting with this task could be considered as a novel activity with many exploratory opportunities, which is still an open area of research for HRI. At the same time, it will be interesting to further investigate what design principles would be applied in developing robots that scaffold children to effectively transit from exploratory actions to intentional behaviors. Individual pace differences of this transition will require for the robot to be adaptively intelligent by dropping old solutions when something shifts in the child's behavior, the task or the context. This demands a dynamic approach of the conceptualization of problem-solving cognitive activity in child-robot cognitive collaboration. Taken together, our results are initial steps toward creating flexible autonomous agents that self-supervise in realistic physical environments by supporting human tendency for self-directed problem-solving activities.

## Data Availability Statement

The datasets generated for this study are available on request to the corresponding author.

## Ethics Statement

The studies involving human participants were reviewed and approved by the committee on the Use of Humans as Experimental Subjects of the Joint Research Centre of the European Commission. Written informed consent to participate in this study was provided by the participants' legal guardian/next of kin and all children assented to participate.

## Author Contributions

VC conceived, designed, executed the study, analyzed the data, and wrote the paper. EG executed the study, analyzed the data, and wrote the paper. LM and GM contributed towards robot development and technical support, executed the study, and wrote the paper. RG overviewed the study, contributed toward robot development, and wrote the paper.

### Conflict of Interest

RG was employed by the Honda Research Institute, Tokyo, Japan, but no commercial or financial relationships were involved for this study. The remaining authors declare that the research was conducted in the absence of any commercial or financial relationships that could be construed as a potential conflict of interest.

## Footnotes

^1^https://tla.mpi.nl/tools/tla-tools/elan/

^2^Values in this study are *O*_3_ = 7, *O*_4_ = 15, *O*_5_ = 31, *O*_6_ = 63, *O*_7_ = 127.
